# IgA nephropathy in adults—treatment standard

**DOI:** 10.1093/ndt/gfad146

**Published:** 2023-07-07

**Authors:** Patrick J Gleeson, Michelle M O'Shaughnessy, Jonathan Barratt

**Affiliations:** Department of Renal Medicine, Cork University Hospital, Cork, Ireland; Department of Medicine, School of Microbiology, APC Microbiome Ireland, University College Cork, Cork, Ireland; Department of Nephrology, Galway University Hospitals, Galway, Ireland; The Mayer IgA Nephropathy Laboratories, University of Leicester, Leicester, UK

**Keywords:** diagnosis, IgA nephropathy, pathophysiology, prognosis, treatment

## Abstract

Immunoglobulin A nephropathy (IgAN) is the most common primary form of glomerular disease worldwide and carries a high lifetime risk of kidney failure. The underlying pathogenesis of IgAN has been characterized to a sub-molecular level; immune complexes containing specific *O*-glycoforms of IgA1 are central. Kidney biopsy remains the gold-standard diagnostic test for IgAN and histological features (i.e. MEST-C score) have also been shown to independently predict outcome. Proteinuria and blood pressure are the main modifiable risk factors for disease progression. No IgAN-specific biomarker has yet been validated for diagnosis, prognosis or tracking response to therapy.

There has been a recent resurgence of investigation into IgAN treatments. Optimized supportive care with lifestyle interventions and non-immunomodulatory drugs remains the backbone of IgAN management. The menu of available reno-protective medications is rapidly expanding beyond blockade of the renin–angiotensin–aldosterone system to include sodium-glucose cotransporter 2 and endothelin type A receptor antagonism. Systemic immunosuppression can further improve kidney outcomes, although recent randomized controlled trials have raised concerns regarding infectious and metabolic toxicity from systemic corticosteroids. Studies evaluating more refined approaches to immunomodulation in IgAN are ongoing: drugs targeting the mucosal immune compartment, B-cell promoting cytokines and the complement cascade are particularly promising.

We review the current standards of treatment and discuss novel developments in pathophysiology, diagnosis, outcome prediction and management of IgAN.

IN A NUTSHELL

1. Immunoglobulin A nephropathy (IgAN) is an immune-mediated glomerular disease with a high lifetime risk of kidney failure and shortened life-expectancy.
2. Kidney-protective therapies, including lifestyle interventions and renin–angiotensin–aldosterone system inhibition, form the cornerstones of optimized supportive care. Sodium-glucose cotransporter 2 inhibition and endothelin receptor antagonism are becoming established as adjunctive reno-protective drugs in IgAN.
3. Prescribing immunosuppressive therapies for patients who remain at high risk of progression to kidney failure ($ \ge $0.75–1 g proteinuria/day) despite optimized supportive care should involve careful weighing of expected risks and benefits, considering patient characteristics and preferences.
4. Corticosteroids are currently the most widely used immunosuppressive therapy for IgAN. Recent studies have reported efficacy of lower doses of systemic corticosteroids accompanied by anti-microbial prophylaxis although treatment emergent toxicity and poor tolerability remain significant concerns even at these lower doses. Targeting corticosteroid delivery to the ileum offers a novel approach that achieves efficacy while reducing systemic toxicity.
5. New insights into the role of the gut mucosal immune system and B-cell promoting cytokines in disease pathogenesis, as well as involvement of the alternative and lectin complement pathways in glomerular injury, continue to inform the development of novel therapeutic approaches for IgAN.

## INTRODUCTION

Immunoglobulin A nephropathy (IgAN) is the most common primary cause of glomerulonephritis worldwide [1, 2]. It has been traditionally thought that IgAN is an indolent disease, which for most does not result in kidney failure during a patient's lifetime. However, median kidney survival from time of IgAN diagnosis was 11.4 years in a large population-based UK cohort [3].

In a Japanese cohort, 50% of patients progressed to kidney failure within 30 years of diagnosis [4].

Disease incidence follows a gradient from West to East, being highest in Japan and China [1]. Patients with Asian-Pacific ancestry experience a more severe disease phenotype [2, 5]. Median age at diagnosis is approximately 40 years old [2, 6] and 15% of patients in a large European cohort were diagnosed in childhood [7], with implications for impaired life participation [8, 9] and shortened life-expectancy [10].

**Figure 1:**
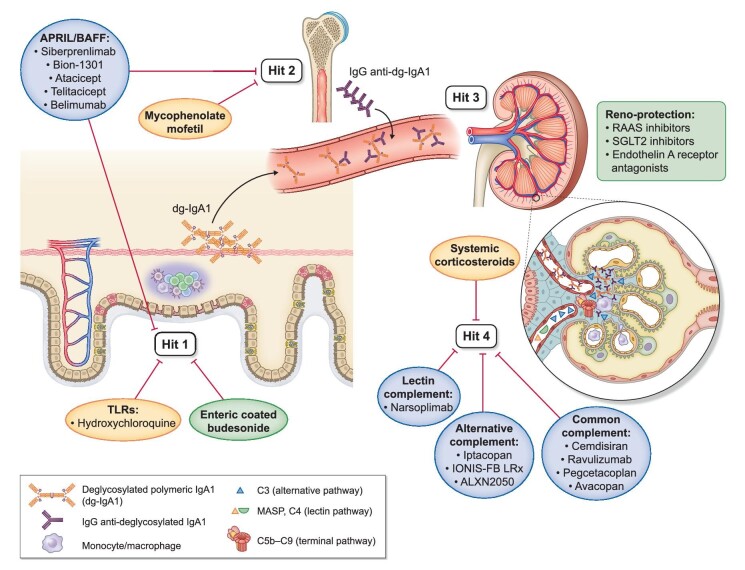
The four pathogenic hits of IgAN targeted by current and new drugs. Hit 1: excessive generation of galactose-deficient IgA1 (gd-IgA1), which enters the circulation from mucosal or possibly systemic sources. Hit 2: generation of auto-reactive IgG autoantibodies that recognise specific *O*-glycoforms of IgA1 (anti-gd-IgA1 IgG). Hit 3: formation of IgA-containing immune complexes in the circulation. Hit 4: deposition of these circulating immune complexes in the glomerular mesangium, triggering an inflammatory cascade that results in mesangial cell proliferation, activation of the alternative and lectin complement pathways, recruitment of monocyte/macrophages and recruitment of T cells from the circulation. Drugs that have been approved by regulatory authorities specifically for the treatment of IgAN are indicated on a green background; drugs currently available in clinical practice but not approved specifically for treatment of IgAN are indicated on an orange background; drugs currently being evaluated in clinical trials for the treatment of IgAN are indicated on a blue background. Hits targeted by specific drugs are indicated by red bars. Reno-protective non-immunomodulatory drugs used to treat IgAN are listed in a box beside the kidney.

IgAN is characterized by IgA dominant, or co-dominant, mesangial immune-complex deposition [11]. Pathogenesis has been shown to involve four main ‘hits’. Hit 1: excess generation of IgA1 with *O*-glycans in the hinge-region deficient in galactose (galactose-deficient IgA1; gd-IgA1); Hit 2: production of anti-gd-IgA1 autoantibodies; Hit 3: formation of IgA1-containing immune complexes; and Hit 4: mesangial deposition of these immune complexes, triggering a cascade of glomerular inflammation culminating in progressive loss of kidney function (Fig. 1) [12].

General kidney-protective measures, including lifestyle modification and inhibition of the renin–angiotensin–aldosterone system (RAASi), are cornerstones of IgAN therapy [13]. Studies examining a role for immunosuppression have, until recently, produced disappointing or conflicting results. More refined approaches to immunosuppression with less toxicity, including targeting of specific molecular pathways, are bringing new hope.

## TREATMENT STANDARDS

The Kidney Disease: Improving Global Outcomes (KDIGO) clinical practice guideline for the management of IgAN was last updated in October 2021 [13]. Rare variants of IgAN including minimal change disease (MCD) with IgA deposition, rapidly progressive IgAN and secondary forms of IgAN, are handled as distinct disease entities in KDIGO guidelines, and are not included in this review, which will focus on typical primary IgAN in adults.

Kidney biopsy is required to make a diagnosis of IgAN. While blood pressure, proteinuria and kidney function primarily determine prognosis, addition of the MEST-C histological score can improve outcome prediction [14]. The KDIGO guidelines recommend that in adults the International IgAN Prediction Tool (IIgANPT) [15], which incorporates the MEST-C score, be applied at the time of kidney biopsy to facilitate prognostication. Variants of this tool have also been validated for use in children [16], and during follow-up [17]. However, consensus regarding how best to apply components of the MEST-C score to guide decisions around drug therapy is lacking. As such, the role of repeat biopsy to guide treatment decisions is limited, unless the disease follows an unexpected course or development of another kidney disease is suspected.

Proteinuria and estimated glomerular filtration rate (eGFR) are the only validated biomarkers, early and late respectively, for kidney failure prognostication and assessment of treatment efficacy in IgAN. Compared with patients with time-averaged proteinuria of <0.5 g/day, those with 0.5–1.0 g/day have a 9.1-fold, and those with >1.0 g/day a 46.5-fold increased hazard of kidney disease progression [18]. Trial-level analyses have concluded that change in proteinuria in response to treatment is a valid surrogate endpoint, which associates closely with clinically important outcomes such as kidney failure [19]. Recent acceptance of proteinuria as a reasonably likely surrogate biomarker of progression to kidney failure by the US Food and Drug Administration (FDA) and European Medicines Agency (EMA) has been a strong stimulus for drug development in IgAN.

Optimized supportive care is recommended as the mainstay of therapy in IgAN [13]. This includes lifestyle modification to incorporate exercise, weight management, smoking cessation, sodium restriction and cardiovascular risk reduction. Blood pressure should be controlled to a target of below 120–130 mmHg systolic and 80 mmHg diastolic, as per general glomerular disease guidelines, with prioritization of RAASi. For patients with proteinuria >0.5 g/day, even without hypertension, introduction of a RAASi is also recommended [20–23].

The Dapagliflozin in Patients with Chronic Kidney Disease (DAPA-CKD) [24] and Empagliflozin in Patients with Chronic Kidney Disease (EMPA-Kidney) [25] studies clearly document the reno-protective effects of sodium-glucose cotransporter 2 inhibitors (SGLT2i) in proteinuric chronic kidney disease (CKD), and these effects have been confirmed by meta-analyses [26, 27]. In EMPA-Kidney, half of the non-diabetic patients with glomerular disease had IgAN, making up 12% of the overall study population, and effect-modification by cause of kidney disease was not observed [28]. A pre-specified subgroup analysis of 270 patients with IgAN in the DAPA-CKD trial showed a reduction in the primary endpoint of sustained eGFR decline ≥50%, kidney failure or death from a kidney or cardiac cause [hazard ratio (HR) 0.29, 95% confidence interval (CI) 0.12–0.73]; a 26% mean reduction in albuminuria was also observed [29]. However, as a caveat to these findings, the primary outcome occurred in the placebo group of DAPA-CKD at over double the anticipated rate, which could be explained by the absence of a run-in period involving optimization of RAASi, as would be typical for IgAN-dedicated studies. Accordingly, while SGLT2i have entered standard clinical care for management of proteinuric kidney disease, the true extent of their additional benefit in IgAN, above optimized supportive care, remains to be quantified.

**Figure 2:**
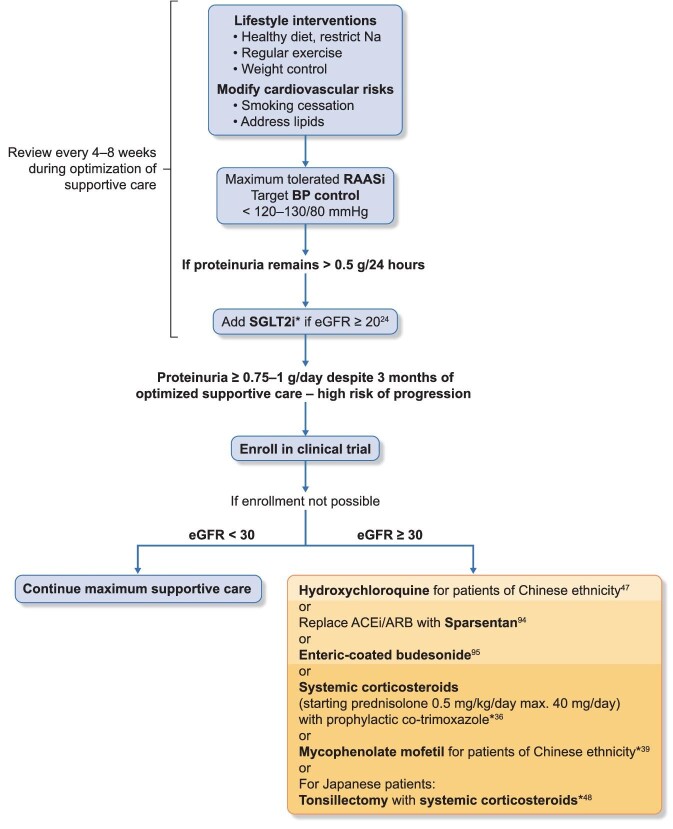
Proposed algorithm for the management of patients with primary IgAN. Lifestyle interventions should include advice on dietary sodium restriction (<2 g sodium/day), smoking cessation, weight control and exercise, as appropriate. Assess cardiovascular risk and commence appropriate interventions. Patients should be reviewed regularly (every 4–8 weeks) during optimization of supportive care. For patients with secondary causes of IgAN, treatment should be targeted at the primary disease. Increasing relative risk of treatment-related toxicity for interventions beyond supportive care is indicated by increasing intensity of the background orange colour in the text box. For risk assessment, see also Box ‘Strategies for personalizing treatment in IgAN’. *The safety of co-administrating SGLT2i with immunosuppression is currently unknown.

Patients who remain at high risk of progression to kidney failure (i.e. with proteinuria ≥0.75–1.0 g/day despite 3 months of optimized supportive care) should ideally be enrolled in an IgAN-focused clinical trial—if this is not feasible or available, then immunosuppressive treatment, typically with corticosteroids, should be considered [13] (Table 1, Fig. 2).

**Table 1:** Drug therapies commonly used and/or approved for the treatment of primary IgAN.

**Drug**	**Dose**	**Marker of response**	**Precautions**	**Toxicity**
RAASi	ACEi: e.g. enalapril 5 mg OD PO uptitrated to 40 mg/day as tolerated [20]ARB: e.g. valsartan 80 mg OD PO uptitrated to 160 mg/day as tolerated [21]	Reduction in proteinuriaReduction in BPReduced eGFR slope	Hold during acute illness (e.g. dehydration, infection)Check for a rise in serum creatinine >30% or hyperkalemia 7–10 days after starting, particularly in patients with an eGFR <30 mL/min/1.73 m^2^ or history of hyperkalemia	HyperkalemiaHypotensionAKI (e.g. in setting of bilateral renal artery stenosis, volume depletion, sepsis)
SGLT2i	Dapagliflozin 10 mg OD PO (DAPA-CKD) [23]Empagliflozin 10 mg OD PO (EMPA-Kidney) [24]	Reduction in proteinuriaReduction in BPReduced eGFR slope	Hold during acute illness (e.g. dehydration, infection)Caution in patients with history of recurrent urinary tract infection or genital *Candida*Efficacy and safety not formally tested in the setting of simultaneous use of immunosuppressants	Genital fungal infectionUrinary tract infectionEuglycaemic diabetic ketoacidosis
Dual ERA/ARB	Sparsentan 200 mg OD PO uptitrated to 400 mg OD PO as tolerated [94]Stop other ACEi/ARB	Reduction in BPReduction in proteinuria	Hold during acute illness (e.g. dehydration, infection)Check for a rise in serum creatinine >30% or hyperkalemia 7–10 days after starting	HyperkalemiaHypotensionAKI (e.g. in setting of bilateral renal artery stenosis, volume depletion, sepsis)Oedema
Enteric-coated budesonide	Budesonide 16 mg OD PO [95]	Reduction in proteinuria	Cautious use in patients with obesity or DM	Glucose intolerance/DMWeight gainAcneAltered appearance (moon facies/hirsutism)
Systemic corticosteroids	Methylprednisolone PO0.4 mg/kg/day max 32 mg/day for 2 months followed by 4 mg/day taper each month for total 6–9 months(TESTING-2 protocol) [36](or prednisolone equivalent)	Reduction in proteinuriaResolution of haematuriaReduced eGFR slope	PJP prophylaxisScreen for and treat prevalent chronic infections (e.g. TB, HBV) prior to initiationCautious use in patients with obesity or DMCautious use in patients with psychiatric historySee ‘Strategies for personalizing treatment in IgAN’	Glucose intolerance/DMOpportunistic infectionWeight gainOsteoporosis/avascular necrosisAcneAltered appearance (stretch marks/buffalo hump/moon facies/hirsutism)Mood/psychosis
MMF	MMF 1.5 g/day for 12 months then taper to 0.75–1 g/day for up to 2 years (MAIN protocol) [39]	Reduction in proteinuriaResolution of haematuriaReduced eGFR slope	Screen for and treat prevalent chronic infections (e.g. TB, HBV) prior to initiationVZV vaccination	DiarrhoeaPneumoniaShinglesLeucopenia

ACEi = angiotensin-converting enzyme inhibitor; ARB = angiotensin-II receptor blockers; OD = once per day; PO = administer orally; AKI = acute kidney injury; TB = tuberculosis; HBV = hepatitis B virus; PJP = *Pneumocystis jirovecii* pneumonia; VZV = Varicella-Zoster virus; M = male; F = female; BP = blood pressure; IS = immunosuppression; IV = intravenously; PCR = protein-creatinine ratio; OR = odds ratio.

The landscape of corticosteroid treatment in IgAN is evolving. Historically, studies of systemic corticosteroids have shown renal benefit [30–33], but were critiqued for their inconsistent use of RAASi and unexpectedly low reporting of treatment-related toxicity. These concerns motivated the more rigorously designed Intensive Supportive Care plus Immunosuppression in IgAN (STOP-IgAN) study, which included a 6 month run-in period to ensure optimization of supportive care prior to randomization [34]. Exclusively European participants, with 0.75–3.0 g proteinuria/day after the run-in phase, were then randomized to supportive care alone or supportive care plus immunosuppression, which comprised corticosteroids for all, with addition of cyclophosphamide followed by azathioprine if eGFR was between 30 and 59 mL/min/1.73 m^2^. While the addition of immunosuppression significantly reduced proteinuria and increased rates of IgAN remission, no difference in eGFR decline was observed between the two groups, even after 10 years of follow-up [35]. Immunosuppressed patients had higher rates of steroid-related metabolic side effects and a non-significant increased risk of infection.

The Effect of Oral Methylprednisolone on Decline in Kidney Function or Kidney Failure in Patients with IgA Nephropathy Randomised Clinical Trial (TESTING)-1 trial of 0.8 mg/kg/day methylprednisolone, in a predominantly Chinese population, was stopped early due to significantly higher rates of infectious complications. There were 13 infectious events in the methylprednisolone group, including 3 incidences of *Pneumocystis* pneumonia, versus none in the placebo group. Benefit against the primary outcome of 40% eGFR reduction, kidney failure or death due to kidney failure (HR 0.37, 95% CI 0.17–0.85) was observed [36].

Subsequently, the protocol was modified (TESTING-2) by reducing the dose of methylprednisolone to 0.4 mg/kg/day (equivalent to 35–40 mg prednisone in a 70 kg patient) and adding prophylactic sulfamethoxazole–trimethoprim. This demonstrated similar benefit for the same primary outcome (HR 0.27, 95% CI 0.11–0.65) with some mitigation of adverse events [37]. While the risk of severe adverse events in TESTING-1 was 16% for full-dose methylprednisolone, compared with 3% for placebo, this difference was reduced to 5% for lower-dose methylprednisolone vs 3% for placebo in TESTING-2. Overall, four deaths attributed to infection were observed in the methylprednisolone groups (three with full-dose, one with lower-dose corticosteroids), as compared with none in the placebo groups. The baseline patient characteristics and study outcomes for these trials (STOP-IgAN, TESTING) are summarized in Table 2.

**Table 2:** Recent randomized placebo-controlled trials of corticosteroids in IgAN.

	**STOP-IgAN (*n* = 162)**	**TESTING (*n* = 503)**	**NefIgArd (Part A) (*n* = 199)**
**Population**	**Primary IgAN**	**Primary IgAN**	**Primary IgAN**
	**Key exclusion criteria**	**Key exclusion criteria**	**Key exclusion criteria**
	**eGFR:** <30 mL/min/1.73 m^2^	**eGFR:** <20 mL/min/1.73 m^2^	**eGFR:** <35 mL/min/1.73 m^2^
	**Proteinuria:** <0.75 g/day after run-in	**Proteinuria:** <1 g/day	**Proteinuria:** <1 g/day
	**BP: No upper limit**	**BP:** >160/110 mmHg	**BP:** ≥140/90 mmHg
	**Immunosuppression:** any prior systemic IS	**Immunosuppression:** systemic IS treatment within 1 year	**Immunosuppression:** systemic IS treatment within 1 year
	**Kidney biopsy:** crescentic IgAN	**Kidney biopsy:** >50% crescents	**Kidney biopsy:** no limit on crescents
	**Key baseline characteristics**	**Key baseline characteristics (total cohort)**	**Key baseline characteristics**
	**Race:** 100% White	**Race:** 95% Asian, 5% White	**Race:** 12% Asian, 86% White, 2% Other
	**Age:** 44 years (mean)	**Age:** 36 years (median)	**Age:** 44 years (median)
	**Gender M:F: 78:22**	**Gender M:F: 60:40**	**Gender M:F: 68:32**
	**eGFR:** 59 mL/min/1.73 m^2^ (mean)	**eGFR:** 58 mL/min/1.73 m^2^ (median)	**eGFR:** 55 mL/min/1.73 m^2^ (median)
	**Proteinuria:** 1.7 g/day (median)	**Proteinuria:** 2.0 g/day (median)	**Proteinuria:** 2.3 g/day (median)
	**RAASi use: 98% (34% dual blockade) 6-month run-in**	**RAASi use: 99.9% 3-month run-in**	**RAASi use: 98% (5% dual blockade) for 3 months**
	**BP: 125/77 mmHg (mean)**	**BP: 124/80 mmHg (median)**	**BP: 126/78 mmHg (median)**
**Intervention**	eGFR >60: methylprednisolone 1 g daily IV × 3 doses at beginning of Months 1, 3 and 5; prednisolone 0.5 mg/kg alternate days PO for 6 month treatment	**High-dose:** methylprednisolone 0.6–0.8 mg/kg per day PO (max 48 mg/day) tapering by 8 mg/day/month for total treatment 6–8 months (*n* = 136)	16 mg enteric-coated budesonide OD PO for 9 months
	Or	Or	
	eGFR 30–59: cyclophosphamide 1.5 mg/kg/day for 3 months followed by azathioprine 1.5 mg/kg/day from Month 3 to Month 36, plus oral prednisolone 40 mg daily PO tapered to 7.5 mg over 6 months and continued for total 36 months	**Low-dose:** Methylprednisolone 0.4 mg/kg/day (max 32 mg/day) weaning by 4 mg/day/month, with prophylactic co-trimoxazole^a^ (*n* = 121)	
			
**Comparator**	Placebo	Placebo	Placebo
**Outcome** (intervention vs placebo)	**Efficacy: primary outcome** **Full clinical remission^b^ at 3 years**17% vs 5% (OR 4.8, *P* = .01)**Decrease in eGFR by ≥15 mL/min/1.73 m^2^ at 3 years**26% vs 28% (OR 0.89, *P* = .75)	**Efficacy: primary outcome** **40% reduction in eGFR, kidney failure, or death due to kidney disease** **Total cohort**28.8% vs 43.1% (HR 0.53, *P *< .001)**Low-dose cohort**6% vs 17% (HR 0.27, no heterogeneity)	**Efficacy: primary outcome** **Reduction in urinary PCR at 9 months**31% reduction vs 5% reduction (*P* = .0003)
	**Key serious adverse events** **Overall:** 35% vs 26%**Deaths** (*n*): 1 vs 1**Infection^c^:** 10% vs 4%**New DM:** 11% vs 1%	**Key serious adverse events—total cohort (low-dose)** **Overall:** 10.9% vs 2.8% (6% vs 2.5%)**Deaths (*n*):** 6 vs 3 (1 vs 0)**Infection^c^:** 7% vs 1% (4% vs 2%)**New DM:** 0.8% vs 0.0% (2% vs 0%)	**Key serious adverse events** **Overall:** 4% vs 1%**Deaths (*n*):** 0 vs 0**Infection^c^:** 0% vs 0%**New DM:** 2% vs 0%

^a^Prophylactic co-trimoxazole was also given to those randomized to placebo after change to low-dose protocol.

^b^In STOP-IgAN full clinical remission was defined as a urinary protein-creatinine ratio of <0.2 g/g and stable renal function with no more than 5 mL/min/1.73 m^2^ change in eGFR.

^c^Serious infectious adverse events were defined as infections requiring hospitalization in TESTING and NefIgArd; they were not specifically defined in STOP-IgAN.

OD = once per day; PO = administer orally; M = male; F = female; BP = blood pressure; IS = immunosuppression; IV = intravenously; PCR = protein-creatinine ratio; OR = odds ratio.

When considering the use of systemic corticosteroids in IgAN, KDIGO guidelines highlight the increased risk of corticosteroid toxicity in individuals with an eGFR <50 mL/min/1.73 m^2^, and suggest avoiding corticosteroids in patients with an eGFR <30 mL/min/1.73 m^2^, diabetes mellitus (DM), obesity, untreated latent infection (e.g. viral hepatitis, tuberculosis or HIV), active peptic ulcer disease, severe osteoporosis or uncontrolled psychiatric illness [13].

Mycophenolate mofetil (MMF) is also included in the KDIGO guidelines as an alternative to corticosteroids, but use should be restricted to Chinese patients, in whom MMF has been shown to preserve renal function and enable reduced corticosteroid exposure in randomized controlled trials (RCTs) [38] with long-term follow-up [39]. A recently published non-blinded RCT from China has confirmed these findings [40]. While infection risk appears lower than with corticosteroids, promoting MMF use as a steroid-sparing strategy, higher rates of pneumonia and herpes zoster when compared with placebo have been reported [40, 41]. To date, studies of MMF in Caucasian patients with IgAN have failed to show benefit, although these studies were small [42, 43] and, in one, the majority of patients had advanced kidney fibrosis at enrolment [44].

The KDIGO guidelines also suggest hydroxychloroquine (HCQ)—an anti-antimalarial that inhibits endosomal Toll-like Receptors (TLRs)—as another option in Chinese patients. Demonstration that TLR7 and TLR9 activation promotes production of nephritogenic IgA provides biological rationale for its therapeutic role [45, 46]. Use of HCQ in Chinese patients is supported by both observational and RCT data [47, 48]; however, HCQ did not show any additional benefit when added to systemic immunosuppression [47]. Data for HCQ are not yet available for Caucasian patients.

The phenomenon of synpharyngitic haematuria in IgAN has motivated tonsillectomy as a therapeutic intervention, particularly in Japan. A meta-analysis of 19 studies showed evidence of benefit in IgAN, but most studies included were observational and only one included Caucasians [49]. A propensity-score matched analysis of European patients from the Validation Study of the Oxford Classification of IgA Nephropathy (VALIGA) cohort found no advantage to tonsillectomy [50]. These findings are reflected in a KDIGO recommendation against performing tonsillectomy for IgAN in Caucasian patients.

Other conventional immunosuppressants, including rituximab, cyclophosphamide and calcineurin inhibitors, have failed to show clinical benefit in IgAN [13]. Multiple other immunomodulatory approaches are currently being evaluated in IgAN (see section ‘New developments’).

**STRATEGIES FOR PERSONALIZING TREATMENT IN IgA NEPHROPATHY**

**Optimized supportive care:** All patients with IgAN should receive individualized optimization of supportive care before considering immunosuppressive therapy. This includes lifestyle modification to incorporate a healthy diet, exercise, weight management, smoking cessation, sodium restriction, blood-pressure management and cardiovascular risk reduction. All patients with >0.5 g proteinuria/day should be offered treatment with RAASi and SGLT2i.

**Considerations when deciding whether to use systemic corticosteroids:** In patients with obesity, DM, active psychiatric disease and/or other relative contraindications to systemic corticosteroid therapy, treatment should primarily focus on supportive care over systemic corticosteroid use. Caution is also advised in those patients with an eGFR<50 mL/min/1.73 m^2^, in whom corticosteroid-related adverse events are more frequent.

**Use of systemic corticosteroids or other immunosuppressive therapies in patients with chronic infection (e.g. hepatitis B, tuberculosis):** Appropriate anti-microbial regimens to treat infection and/or prevent reactivation should be initiated before prescribing immunosuppressive therapies. Modified immunosuppression protocols may be considered, in collaboration with infectious disease services, if appropriate pathogen elimination or suppression can be achieved.

**Ethnicity:** In Chinese patients, MMF offers an effective and potentially safer alternative to systemic corticosteroids, although it still carries an increased risk of infectious complications. Use of HCQ is another option for Chinese patients. In Japanese patients, tonsillectomy with concurrent corticosteroids may be considered.

## NEW DEVELOPMENTS

### Pathophysiology

Multiple lines of evidence support a mucosal source of pathogenic IgA in IgAN. Circulating IgA immune complexes and glomerular eluates are enriched for the polymeric, mucosal, form of IgA1 [51–54]. Mesangial deposits of secretory IgA are seen in 15%–30% of IgAN biopsies. Genome-wide association studies have highlighted the mucosal immune system as the principal pathway involved in disease pathogenesis [55]. Mucosal infection [56] is associated with exacerbations of IgAN, while coeliac disease and inflammatory bowel disease are important secondary causes of IgAN [57]. Circulating mucosally primed IgA^+^ B cells, expressing α4β7 and C-C chemokine receptor type 9 (CCR9), are increased in IgAN [58]. Lack of efficacy of rituximab in IgAN may be explained by low CD20 expression on IgA^+^ mucosal plasmablasts [59, 60]. Alterations in the oropharyngeal microbiota have been described in both Asian and European IgAN [61–64]. In the intestinal microbiota, significant differences in microbial diversity and species level abundance have been identified between progressive IgAN, non-progressive IgAN and healthy subjects [65].

T-cell independent mucosal IgA production is orchestrated by cytokines including A Proliferation Inducing Ligand (APRIL) and B-cell Activating Factor (BAFF). The genes encoding APRIL (*TNFSF13*) and one of its receptors (*TACI*) have been identified as IgAN genetic risk loci [66]. Serum levels of both BAFF and APRIL are elevated in IgAN [58, 67–69].

Changes to *O*-glycans in the IgA1 hinge region have long been recognized as a key feature of IgAN [70]. An absence of sialic acid residues has now been shown to be equally, if not more, important than reduction in hinge-region galactose content [71, 72]. *O*-linked *N*-acetylgalactosamine (O-GalNac) residues, exposed by this absence of sialic acid and galactose residues, are the antigenic targets of autoantibodies [73]. The dominant autoantibody isotype is IgG2 [74], which may explain the absence of mesangial C1q in IgAN as, similar to IgA1, IgG2 is not an effective activator of the classical complement pathway [75, 76]. Instead, the alternative and lectin complement pathways are activated [77].

### Diagnosis

Kidney biopsy remains the only way to diagnose IgAN. While average circulating levels of IgA, IgA1, specific IgA1 *O*-glycoforms, anti-glycan autoantibodies and IgA immune complexes are elevated in IgAN, the sensitivity and specificity of these biomarkers is insufficient to allow their use as diagnostic tests [78].

### Outcome prediction

A number of novel serum, urine and kidney prognostic biomarkers have been reported [79]. Most interest has focused on measuring serum levels of gd-IgA1 [80–83] or measuring urinary excretion of epidermal growth factor/monocyte chemotactic peptide-1 ratio [84], CXCL1 [85], soluble CD89 [86] and soluble CD163 [87]. Numerous mesangial markers of complement activation have been reported to predict renal outcome including C4d, mannose binding lectin, complement factor H related proteins 1 and 5, and C3a/C5a [88–91]. However, for all of these serum, urine and tissue biomarkers, it is not known whether their measurement adds prognostic value above that provided by the IIgANPT [15].

### Management

SGLT2 inhibition is becoming established as an important adjunct to optimize supportive care in IgAN. Other agents that may further enhance supportive care include mineralocorticoid receptor antagonists (MRA) [92] and endothelin A receptor antagonists (ERA) [93, 94]. While no studies of MRA have been performed in IgAN, there are two ongoing phase 3 studies of ERAs in IgAN (sparsentan, NCT03762850; and atrasentan, NCT04573478). An interim analysis of the PROTECT study of sparsentan demonstrated a significant reduction in proteinuria at 9 months with half as many patients in the sparsentan group developing a 40% reduction in eGFR compared with the irbesartan group [95]. The US FDA has now approved sparsentan for patients with IgAN at high risk of progressive kidney function decline (Fig. 1, Table 1).

Immunosuppressive therapy in IgAN is often accompanied by an unacceptably high risk of infection. Polymorphisms in genetic loci involved in immune-signalling pathways important for defence against opportunistic pathogens, such as *Pneumocystis*, have been reported in IgAN. This could explain why immunosuppressed IgAN patients are particularly susceptible to infection [96]. In the TESTING-2 study, administration of prophylactic antimicrobials along with a reduction in dose of corticosteroids helped to mitigate against this infection risk [37].

Delivery of corticosteroids to the terminal ileum is a novel way of directly targeting the gut-associated lymphoid tissue (GALT), while minimizing systemic metabolic and infectious complications. In Part A of the Effect of Nefecton in Patients with Primary IgA Nephropathy at Risk of Developing End-Stage Renal Disease (NefIgArd) study, 199 patients at high-risk of progression despite optimized supportive care were randomized (1:1) to receive 16 mg of enteric-coated budesonide or placebo for 9 months. The intervention group achieved 48% greater reduction in proteinuria by 1 year compared with placebo [97], similar to the reduction in proteinuria seen after 1 year with systemic corticosteroids in the TESTING studies [37] (Table 2). Based on these data, enteric-coated budesonide became the first drug approved by both the US FDA and EMA for treatment of patients with IgAN at high risk of progressive kidney function decline. A commercial press release has reported promising results for Part B of the study, looking at 2-year eGFR data for the full NefIgArd cohort (*n* = 360)—peer-reviewed results are eagerly awaited [97]. Adverse effects reported in Part A were consistent with some systemic budesonide absorption (approximately 10% of the delivered dose) and included higher rates of hypertension, oedema and acne in the treatment arm. However, there was no increased risk of infection. This novel therapeutic approach has the potential to target a major site of pathogenic IgA production in IgAN, while minimizing systemic toxicity (Fig. 1, Table 1).

Targeting BAFF and/or APRIL signalling is another promising approach. Current therapies under investigation include monoclonal antibodies specific for APRIL (Bion-1301, NCT03945318; and sibeprenlimab, NCT05248646) or BAFF (belimumab, EudraCT 2017-004366-10), and decoy TACI (transmembrane activator and CAML interactor) receptors for BAFF/APRIL (atacicept, NCT04716231; and telitacicept, NCT04905212). Early data from phase 2 studies have shown that these approaches reduce circulating levels of gd-IgA1 and also significantly reduce proteinuria [98–100]. A phase 3 study of siberprenlimab is ongoing, with phase 3 studies of other drugs interrupting this pathway planned to start in 2023 (Fig. 1).

A plethora of drugs inhibiting different stages of the complement cascade are also under investigation [101]. Approaches have targeted proteins of the terminal complement pathway, including C3 (pegcetacoplan, NCT03453619), C5 (cemdisiran, NCT03841448; and ravulizumab, NCT04564339) and the C5a receptor (avacopan, NCT02384317). Early data suggest that terminal pathway inhibition can reduce proteinuria in IgAN [102, 103]. Phase 2 and 3 studies of drugs targeting factor B (iptacopan, NCT04578834; and IONIS-FB-LRx, NCT04014335) and factor D (ALXN2050, NCT05097989) of the alternative pathway, and MASP-2 (narsoplimab, NCT03608033) of the lectin pathway are also underway—early results again support their beneficial impact on proteinuria [104, 105] (Fig. 1).

Significant heterogeneity in clinical, epidemiological and immunological aspects suggest the characteristic glomerular lesions of IgAN might represent a common final pathway of a spectrum of disease endotypes. Variation in underlying disease processes could result in different responses to treatment between individuals and across populations, particularly for more targeted immunotherapies. The optimal timing of initiation and duration of immunotherapies for IgAN also remain to be defined.

## SUMMARY

Recent data from the largest available global IgAN registry demonstrate a considerable lifetime risk of kidney failure in IgAN [3]. The slowly progressive nature of IgAN should not lull the nephrologist, or patient, into a false sense of security and inaction.

Goal-directed optimized supportive care should be delivered to all patients with IgAN—available drug options are expanding, with the addition of SGLT2i and recent approval of the first ERA for use in IgAN. It is, however, important to acknowledge that these approaches do nothing to prevent IgA immune complex formation and mesangial deposition. Accordingly, immunomodulatory and anti-inflammatory therapies are also needed in many cases to suppress pathogenic IgA production and control glomerular inflammation. Until recently, there were few, if any, drugs available that are safe, well tolerated and capable of modifying disease progression. Suppression of pathogenic IgA production through targeting the gut immune system (e.g. enteric-coated budesonide or inhibition of BAFF and/or APRIL signalling) along with treatments to rapidly reduce glomerular inflammation and prevent maladaptive glomerular remodelling (e.g. lower dose systemic corticosteroids or inhibition of glomerular complement activation) are emerging as exciting treatment approaches.

It is our opinion that to prevent kidney failure during the lifetime of a patient with IgAN, multi-targeted combination therapies will be required. Treatment will need to be commenced early, with supportive and disease-modifying therapies used simultaneously. To better inform personalized patient management, and to monitor therapeutic response, we are in desperate need of validated biomarkers—this must be the focus of future research.

## Supplementary Material

gfad146_Supplemental_File

## Data Availability

No new data were generated or analysed in support of this research.

## References

[1] McGrogan A, Franssen CF, de Vries CS. The incidence of primary glomerulonephritis worldwide: a systematic review of the literature. Nephrol Dial Transplant 2011;26:414–30. 10.1093/ndt/gfq665 21068142

[2] Schena FP, Nistor I. Epidemiology of IgA nephropathy: a global perspective. Semin Nephrol 2018;38:435–42. 10.1016/j.semnephrol.2018.05.013 30177015

[3] Pitcher D, Braddon F, Hendry B et al. Long-term outcomes in IgA nephropathy. Clin J Am Soc Nephrol 2023;18:727–38. 10.2215/CJN.0000000000000135 37055195 PMC10278810

[4] Moriyama T, Tanaka K, Iwasaki C et al. Prognosis in IgA nephropathy: 30-year analysis of 1,012 patients at a single center in Japan. PLoS One 2014;9:e91756. 10.1371/journal.pone.0091756 24658533 PMC3962373

[5] Barbour SJ, Cattran DC, Kim SJ et al. Individuals of Pacific Asian origin with IgA nephropathy have an increased risk of progression to end-stage renal disease. Kidney Int 2013;84:1017–24. 10.1038/ki.2013.210 23739233

[6] D'Amico G . Natural history of idiopathic IgA nephropathy and factors predictive of disease outcome. Semin Nephrol 2004;24:179–96. 10.1016/j.semnephrol.2004.01.001 15156525

[7] Coppo R, Troyanov S, Bellur S et al. Validation of the Oxford classification of IgA nephropathy in cohorts with different presentations and treatments. Kidney Int 2014;86:828–36. 10.1038/ki.2014.63 24694989 PMC4184028

[8] Kerklaan J, Hannan E, Hanson C et al. Perspectives on life participation by young adults with chronic kidney disease: an interview study. BMJ Open 2020;10:e037840. 10.1136/bmjopen-2020-037840 PMC7569939 33067282

[9] Carter SA, Gutman T, Logeman C et al. Identifying outcomes important to patients with glomerular disease and their caregivers. Clin J Am Soc Nephrol 2020;15:673–84. 10.2215/CJN.13101019 32354728 PMC7269216

[10] Jarrick S, Lundberg S, Welander A et al. Mortality in IgA nephropathy: a nationwide population-based cohort study. J Am Soc Nephrol 2019;30:866–76. 10.1681/ASN.2018101017 30971457 PMC6493992

[11] Wyatt RJ, Julian BA. IgA nephropathy. N Engl J Med 2013;368:2402–14. 10.1056/NEJMra1206793 23782179

[12] Suzuki H, Kiryluk K, Novak J et al. The pathophysiology of IgA nephropathy. J Am Soc Nephrol 2011;22:1795–803. 10.1681/ASN.2011050464 21949093 PMC3892742

[13] Rovin BH, Adler SG, Barratt J et al. Executive summary of the KDIGO 2021 Guideline for the management of glomerular diseases. Kidney Int 2021;100:753–79. 10.1016/j.kint.2021.05.015 34556300

[14] Barbour SJ, Espino-Hernandez G, Reich HN et al. The MEST score provides earlier risk prediction in lgA nephropathy. Kidney Int 2016;89:167–75. 10.1038/ki.2015.322 26759049

[15] Barbour SJ, Coppo R, Zhang H et al. Evaluating a new international risk-prediction tool in IgA nephropathy. JAMA Intern Med 2019;179:942–52. 10.1001/jamainternmed.2019.0600 30980653 PMC6583088

[16] Barbour SJ, Coppo R, Er L et al. Updating the International IgA Nephropathy Prediction Tool for use in children. Kidney Int 2021;99:1439–50. 10.1016/j.kint.2020.10.033 33220356

[17] Barbour SJ, Coppo R, Zhang H et al. Application of the International IgA Nephropathy Prediction Tool one or two years post-biopsy. Kidney Int 2022;102:160–72. 10.1016/j.kint.2022.02.042 35490842

[18] Le W, Liang S, Hu Y et al. Long-term renal survival and related risk factors in patients with IgA nephropathy: results from a cohort of 1155 cases in a Chinese adult population. Nephrol Dial Transplant 2012;27:1479–85. 10.1093/ndt/gfr527 21965586

[19] Thompson A, Carroll K, Inker LA et al. Proteinuria reduction as a surrogate end point in trials of IgA nephropathy. Clin J Am Soc Nephrol 2019;14:469–81. 10.2215/CJN.08600718 30635299 PMC6419287

[20] Reich HN, Troyanov S, Scholey JW et al. Remission of proteinuria improves prognosis in IgA nephropathy. J Am Soc Nephrol 2007;18:3177–83. 10.1681/ASN.2007050526 17978307

[21] Praga M, Gutierrez E, Gonzalez E et al. Treatment of IgA nephropathy with ACE inhibitors: a randomized and controlled trial. J Am Soc Nephrol 2003;14:1578–83. 10.1097/01.ASN.0000068460.37369.DC 12761258

[22] Li PK, Leung CB, Chow KM et al. Hong Kong study using valsartan in IgA nephropathy (HKVIN): a double-blind, randomized, placebo-controlled study. Am J Kidney Dis 2006;47:751–60. 10.1053/j.ajkd.2006.01.017 16632013

[23] Nakamura T, Ushiyama C, Suzuki S et al. Effects of angiotensin-converting enzyme inhibitor, angiotensin II receptor antagonist and calcium antagonist on urinary podocytes in patients with IgA nephropathy. Am J Nephrol 2000;20:373–9. 10.1159/000013619 11092994

[24] Heerspink HJL, Stefansson BV, Correa-Rotter R et al. Dapagliflozin in patients with chronic kidney disease. N Engl J Med 2020;383:1436–46. 10.1056/NEJMoa2024816 32970396

[25] The E-KCG, Herrington WG, Staplin N et al. Empagliflozin in patients with chronic kidney disease. N Engl J Med 2023;388:117–27. 36331190 10.1056/NEJMoa2204233 PMC7614055

[26] Nuffield Department of Population Health Renal Studies Group; SGLT2 inhibitor Meta-Analysis Cardio-Renal Trialists’ Consortium . Impact of diabetes on the effects of sodium glucose co-transporter-2 inhibitors on kidney outcomes: collaborative meta-analysis of large placebo-controlled trials. Lancet 2022;400:1788–801. 10.1016/S0140-6736(22)02074-8 36351458 PMC7613836

[27] Braunwald E . Gliflozins in the management of cardiovascular disease. N Engl J Med 2022;386:2024–34. 10.1056/NEJMra2115011 35613023

[28] EMPA-KIDNEY Collaborative Group . Design, recruitment, and baseline characteristics of the EMPA-KIDNEY trial. Nephrol Dial Transplant 2022;37:1317–29. 10.1093/ndt/gfac040 35238940 PMC9217655

[29] Wheeler DC, Toto RD, Stefansson BV et al. A pre-specified analysis of the DAPA-CKD trial demonstrates the effects of dapagliflozin on major adverse kidney events in patients with IgA nephropathy. Kidney Int 2021;100:215–24. 10.1016/j.kint.2021.03.033 33878338

[30] Pozzi C, Andrulli S, Del Vecchio L et al. Corticosteroid effectiveness in IgA nephropathy: long-term results of a randomized, controlled trial. J Am Soc Nephrol 2004;15:157–63. 10.1097/01.ASN.0000103869.08096.4F 14694168

[31] Pozzi C, Bolasco PG, Fogazzi GB et al. Corticosteroids in IgA nephropathy: a randomised controlled trial. Lancet 1999;353:883–7. 10.1016/S0140-6736(98)03563-6 10093981

[32] Manno C, Torres DD, Rossini M et al. Randomized controlled clinical trial of corticosteroids plus ACE-inhibitors with long-term follow-up in proteinuric IgA nephropathy. Nephrol Dial Transplant 2009;24:3694–701. 10.1093/ndt/gfp356 19628647

[33] Lv J, Zhang H, Chen Y et al. Combination therapy of prednisone and ACE inhibitor versus ACE-inhibitor therapy alone in patients with IgA nephropathy: a randomized controlled trial. Am J Kidney Dis 2009;53:26–32. 10.1053/j.ajkd.2008.07.029 18930568

[34] Rauen T, Eitner F, Fitzner C et al. Intensive supportive care plus immunosuppression in IgA nephropathy. N Engl J Med 2015;373:2225–36. 10.1056/NEJMoa1415463 26630142

[35] Rauen T, Wied S, Fitzner C et al. After ten years of follow-up, no difference between supportive care plus immunosuppression and supportive care alone in IgA nephropathy. Kidney Int 2020;98:1044–52. 10.1016/j.kint.2020.04.046 32450154

[36] Lv J, Zhang H, Wong MG et al. Effect of oral methylprednisolone on clinical outcomes in patients with IgA nephropathy: the TESTING randomized clinical trial. JAMA 2017;318:432–42. 10.1001/jama.2017.9362 28763548 PMC5817603

[37] Lv J, Wong MG, Hladunewich MA et al. Effect of oral methylprednisolone on decline in kidney function or kidney failure in patients with IgA nephropathy: the TESTING randomized clinical trial. JAMA 2022;327:1888–98. 10.1001/jama.2022.5368 35579642 PMC9115617

[38] Tang S, Leung JC, Chan LY et al. Mycophenolate mofetil alleviates persistent proteinuria in IgA nephropathy. Kidney Int 2005;68:802–12. 10.1111/j.1523-1755.2005.00460.x 16014059

[39] Tang SC, Tang AW, Wong SS et al. Long-term study of mycophenolate mofetil treatment in IgA nephropathy. Kidney Int 2010;77:543–9. 10.1038/ki.2009.499 20032964

[40] Hou FF, Xie D, Wang J et al. Effectiveness of mycophenolate mofetil among patients with progressive IgA nephropathy: a randomized clinical trial. JAMA Netw Open 2023;6:e2254054. 10.1001/jamanetworkopen.2022.54054 36745456 PMC12578496

[41] Hou JH, Le WB, Chen N et al. Mycophenolate mofetil combined with prednisone versus full-dose prednisone in IgA nephropathy with active proliferative lesions: a randomized controlled trial. Am J Kidney Dis 2017;69:788–95. 10.1053/j.ajkd.2016.11.027 28215945

[42] Hogg RJ, Bay RC, Jennette JC et al. Randomized controlled trial of mycophenolate mofetil in children, adolescents, and adults with IgA nephropathy. Am J Kidney Dis 2015;66:783–91. 10.1053/j.ajkd.2015.06.013 26209543

[43] Maes BD, Oyen R, Claes K et al. Mycophenolate mofetil in IgA nephropathy: results of a 3-year prospective placebo-controlled randomized study. Kidney Int 2004;65:1842–9. 10.1111/j.1523-1755.2004.00588.x 15086925

[44] Frisch G, Lin J, Rosenstock J et al. Mycophenolate mofetil (MMF) vs placebo in patients with moderately advanced IgA nephropathy: a double-blind randomized controlled trial. Nephrol Dial Transplant 2005;20:2139–45. 10.1093/ndt/gfh974 16030050

[45] Zheng N, Xie K, Ye H et al. TLR7 in B cells promotes renal inflammation and Gd-IgA1 synthesis in IgA nephropathy. JCI Insight 2020;5:e136965. 10.1172/jci.insight.136965 PMC7453916 32699192

[46] Makita Y, Suzuki H, Kano T et al. TLR9 activation induces aberrant IgA glycosylation via APRIL- and IL-6-mediated pathways in IgA nephropathy. Kidney Int 2020;97:340–9. 10.1016/j.kint.2019.08.022 31748116 PMC7372907

[47] Stefan G, Mircescu G. Hydroxychloroquine in IgA nephropathy: a systematic review. Ren Fail 2021;43:1520–7. 10.1080/0886022X.2021.2000875 34779707 PMC8604447

[48] Liu LJ, Yang YZ, Shi SF et al. Effects of hydroxychloroquine on proteinuria in IgA nephropathy: a randomized controlled trial. Am J Kidney Dis 2019;74:15–22. 10.1053/j.ajkd.2019.01.026 30922594

[49] Duan J, Liu D, Duan G et al. Long-term efficacy of tonsillectomy as a treatment in patients with IgA nephropathy: a meta-analysis. Int Urol Nephrol 2017;49:103–12. 10.1007/s11255-016-1432-7 27722990

[50] Feehally J, Coppo R, Troyanov S et al. Tonsillectomy in a European cohort of 1,147 patients with IgA nephropathy. Nephron 2016;132:15–24. 10.1159/000441852 26586175

