# Supplementary material for: IgA nephropathy in adults—treatment standard
Source: Nephrol Dial Transplant. 2023 Jul 7;38(11):2464–73. doi: 10.1093/ndt/gfad146 (PMC10794095; doi:10.1093/ndt/gfad146)
Supplement: gfad146_Supplemental_File [file gfad146_supplemental_file.docx]

**Supplementary References:**

51. Monteiro RC, Halbwachs-Mecarelli L, Roque-Barreira MC*, et al.* Charge and size of mesangial IgA in IgA nephropathy. *Kidney Int* 1985; **28:** 666-671.

52. Emancipator SN, Gallo GR, Lamm ME. Experimental IgA nephropathy induced by oral immunization. *J Exp Med* 1983; **157:** 572-582.

53. Barratt J, Bailey EM, Buck KS*, et al.* Exaggerated systemic antibody response to mucosal Helicobacter pylori infection in IgA nephropathy. *Am J Kidney Dis* 1999; **33:** 1049-1057.

54. Smith AC, Molyneux K, Feehally J*, et al.* O-glycosylation of serum IgA1 antibodies against mucosal and systemic antigens in IgA nephropathy. *J Am Soc Nephrol* 2006; **17:** 3520-3528.

55. Kiryluk K, Li Y, Scolari F*, et al.* Discovery of new risk loci for IgA nephropathy implicates genes involved in immunity against intestinal pathogens. *Nat Genet* 2014; **46:** 1187-1196.

56. Magistroni R, D'Agati VD, Appel GB*, et al.* New developments in the genetics, pathogenesis, and therapy of IgA nephropathy. *Kidney Int* 2015; **88:** 974-989.

57. Ambruzs JM, Walker PD, Larsen CP. The histopathologic spectrum of kidney biopsies in patients with inflammatory bowel disease. *Clin J Am Soc Nephrol* 2014; **9:** 265-270.

58. Sallustio F, Curci C, Chaoul N*, et al.* High levels of gut-homing immunoglobulin A+ B lymphocytes support the pathogenic role of intestinal mucosal hyperresponsiveness in immunoglobulin A nephropathy patients. *Nephrol Dial Transplant* 2021; **36:** 1765.

59. Mei HE, Frolich D, Giesecke C*, et al.* Steady-state generation of mucosal IgA+ plasmablasts is not abrogated by B-cell depletion therapy with rituximab. *Blood* 2010; **116:** 5181-5190.

60. Lafayette RA, Canetta PA, Rovin BH*, et al.* A Randomized, Controlled Trial of Rituximab in IgA Nephropathy with Proteinuria and Renal Dysfunction. *J Am Soc Nephrol* 2017; **28:** 1306-1313.

61. Yamaguchi H, Goto S, Takahashi N*, et al.* Aberrant mucosal immunoreaction to tonsillar microbiota in immunoglobulin A nephropathy. *Nephrol Dial Transplant* 2021; **36:** 75-86.

62. Nagasawa Y, Nomura R, Misaki T*, et al.* Relationship between IgA Nephropathy and Porphyromonas gingivalis; Red Complex of Periodontopathic Bacterial Species. *Int J Mol Sci* 2021; **22**.

63. Piccolo M, De Angelis M, Lauriero G*, et al.* Salivary Microbiota Associated with Immunoglobulin A Nephropathy. *Microb Ecol* 2015; **70:** 557-565.

64. Park JI, Kim TY, Oh B*, et al.* Comparative analysis of the tonsillar microbiota in IgA nephropathy and other glomerular diseases. *Sci Rep* 2020; **10:** 16206.

65. De Angelis M, Montemurno E, Piccolo M*, et al.* Microbiota and metabolome associated with immunoglobulin A nephropathy (IgAN). *PLoS One* 2014; **9:** e99006.

66. Kiryluk K. GWAS defines pathogenic signaling pathways and prioritizes 5 drug targets for IgA nephropathy. *MedRxIV* 2021.

67. Han SS, Yang SH, Choi M*, et al.* The Role of TNF Superfamily Member 13 in the Progression of IgA Nephropathy. *J Am Soc Nephrol* 2016; **27:** 3430-3439.

68. Zhai YL, Zhu L, Shi SF*, et al.* Increased APRIL Expression Induces IgA1 Aberrant Glycosylation in IgA Nephropathy. *Medicine (Baltimore)* 2016; **95:** e3099.

69. Takahara M, Nagato T, Nozaki Y*, et al.* A proliferation-inducing ligand (APRIL) induced hyper-production of IgA from tonsillar mononuclear cells in patients with IgA nephropathy. *Cell Immunol* 2019; **341:** 103925.

70. Novak J, Julian BA, Tomana M*, et al.* IgA glycosylation and IgA immune complexes in the pathogenesis of IgA nephropathy. *Semin Nephrol* 2008; **28:** 78-87.

71. Hiki Y, Odani H, Takahashi M*, et al.* Mass spectrometry proves under-O-glycosylation of glomerular IgA1 in IgA nephropathy. *Kidney Int* 2001; **59:** 1077-1085.

72. Dotz V, Visconti A, Lomax-Browne HJ*, et al.* O- and N-Glycosylation of Serum Immunoglobulin A is Associated with IgA Nephropathy and Glomerular Function. *J Am Soc Nephrol* 2021; **32:** 2455-2465.

73. Rizk DV, Saha MK, Hall S*, et al.* Glomerular Immunodeposits of Patients with IgA Nephropathy Are Enriched for IgG Autoantibodies Specific for Galactose-Deficient IgA1. *J Am Soc Nephrol* 2019; **30:** 2017-2026.

74. Tomana M, Novak J, Julian BA*, et al.* Circulating immune complexes in IgA nephropathy consist of IgA1 with galactose-deficient hinge region and antiglycan antibodies. *J Clin Invest* 1999; **104:** 73-81.

75. Michaelsen TE, Garred P, Aase A. Human IgG subclass pattern of inducing complement-mediated cytolysis depends on antigen concentration and to a lesser extent on epitope patchiness, antibody affinity and complement concentration. *Eur J Immunol* 1991; **21:** 11-16.

76. *Janeway’s Immunobiology*, 10th edn, 2022.

77. Le Stang MB, Gleeson PJ, Daha MR*, et al.* Is complement the main accomplice in IgA nephropathy? From initial observations to potential complement-targeted therapies. *Mol Immunol* 2021; **140:** 1-11.

78. Hastings MC, Moldoveanu Z, Suzuki H*, et al.* Biomarkers in IgA nephropathy: relationship to pathogenetic hits. *Expert Opin Med Diagn* 2013; **7:** 615-627.

79. Selvaskandan H, Shi S, Twaij S*, et al.* Monitoring Immune Responses in IgA Nephropathy: Biomarkers to Guide Management. *Front Immunol* 2020; **11:** 572754.

80. Wada Y, Matsumoto K, Suzuki T*, et al.* Clinical significance of serum and mesangial galactose-deficient IgA1 in patients with IgA nephropathy. *PLoS One* 2018; **13:** e0206865.

81. Maixnerova D, Ling C, Hall S*, et al.* Galactose-deficient IgA1 and the corresponding IgG autoantibodies predict IgA nephropathy progression. *PLoS One* 2019; **14:** e0212254.

82. Martin-Penagos L, Fernandez-Fresnedo G, Benito-Hernandez A*, et al.* Measurement of galactosyl-deficient IgA1 by the monoclonal antibody KM55 contributes to predicting patients with IgA nephropathy with high risk of long-term progression. *Nefrologia (Engl Ed)* 2021; **41:** 311-320.

83. Berthoux F, Suzuki H, Thibaudin L*, et al.* Autoantibodies targeting galactose-deficient IgA1 associate with progression of IgA nephropathy. *J Am Soc Nephrol* 2012; **23:** 1579-1587.

84. Torres DD, Rossini M, Manno C*, et al.* The ratio of epidermal growth factor to monocyte chemotactic peptide-1 in the urine predicts renal prognosis in IgA nephropathy. *Kidney Int* 2008; **73:** 327-333.

85. Hisano S, Joh K, Katafuchi R*, et al.* Reproducibility for pathological prognostic parameters of the Oxford classification of IgA nephropathy: the authors reply. *Clin Exp Nephrol* 2017; **21:** 1137-1138.

86. Cambier A, Gleeson PJ, Abbad L*, et al.* Soluble CD89 is a critical factor for mesangial proliferation in childhood IgA nephropathy. *Kidney Int* 2022; **101:** 274-287.

87. Gong S, Jin S, Li Y*, et al.* Urinary Soluble CD163 Levels Predict IgA Nephropathy Remission Status. *Front Immunol* 2021; **12:** 769802.

88. Espinosa M, Ortega R, Sanchez M*, et al.* Association of C4d deposition with clinical outcomes in IgA nephropathy. *Clin J Am Soc Nephrol* 2014; **9:** 897-904.

89. Roos A, Rastaldi MP, Calvaresi N*, et al.* Glomerular activation of the lectin pathway of complement in IgA nephropathy is associated with more severe renal disease. *J Am Soc Nephrol* 2006; **17:** 1724-1734.

90. Medjeral-Thomas NR, Troldborg A, Constantinou N*, et al.* Progressive IgA Nephropathy Is Associated With Low Circulating Mannan-Binding Lectin-Associated Serine Protease-3 (MASP-3) and Increased Glomerular Factor H-Related Protein-5 (FHR5) Deposition. *Kidney Int Rep* 2018; **3:** 426-438.

91. Liu L, Zhang Y, Duan X*, et al.* C3a, C5a renal expression and their receptors are correlated to severity of IgA nephropathy. *J Clin Immunol* 2014; **34:** 224-232.

92. Bakris GL, Agarwal R, Anker SD*, et al.* Effect of Finerenone on Chronic Kidney Disease Outcomes in Type 2 Diabetes. *N Engl J Med* 2020; **383:** 2219-2229.

93. Heerspink HJL, Parving HH, Andress DL*, et al.* Atrasentan and renal events in patients with type 2 diabetes and chronic kidney disease (SONAR): a double-blind, randomised, placebo-controlled trial. *Lancet* 2019; **393:** 1937-1947.

94. Trachtman H, Nelson P, Adler S*, et al.* DUET: A Phase 2 Study Evaluating the Efficacy and Safety of Sparsentan in Patients with FSGS. *J Am Soc Nephrol* 2018; **29:** 2745-2754.

95. Heerspink HJL, Radhakrishnan J, Alpers CE*, et al.* Sparsentan in patients with IgA nephropathy: a prespecified interim analysis from a randomised, double-blind, active-controlled clinical trial. *Lancet* 2023.

96. Gleeson PJ, Sokol H, Monteiro RC. CARD9, VAV3, and infection risk in IgA nephropathy. *Kidney Int* 2023; **103:** 996-997.

97. Barratt J, Lafayette R, Kristensen J*, et al.* Results from part A of the multi-center, double-blind, randomized, placebo-controlled NefIgArd trial, which evaluated targeted-release formulation of budesonide for the treatment of primary immunoglobulin A nephropathy. *Kidney Int* 2022.

98. Barratt J HB, Schwartz BS, Sorensen B, Roy SE, Stromatt CL, MacDonald M, Endlsey AN, Lo J, Glicklich A, King AJ: Pharmacodynamic and Clinical Responses to BION-1301 in Patients with IgA Nephropathy: Initial Results of a Ph1/2 Trial. In *ASN Kidney Week 2021*, 2021

99. Mathur M, Barratt J, Suzuki Y*, et al.* Safety, Tolerability, Pharmacokinetics, and Pharmacodynamics of VIS649 (Sibeprenlimab), an APRIL-Neutralizing IgG2 Monoclonal Antibody, in Healthy Volunteers. *Kidney Int Rep* 2022; **7:** 993-1003.

100. Lv J, Liu L, Hao C*, et al.* Randomized Phase 2 Trial of Telitacicept in Patients With IgA Nephropathy With Persistent Proteinuria. *Kidney Int Rep* 2023; **8:** 499-506.

101. Cheung CK, Dormer JP, Barratt J. The role of complement in glomerulonephritis - are novel therapies ready for prime time? *Nephrol Dial Transplant* 2022.

102. Bruchfeld A, Magin H, Nachman P*, et al.* C5a receptor inhibitor avacopan in immunoglobulin A nephropathy-an open-label pilot study. *Clin Kidney J* 2022; **15:** 922-928.

103. Barratt J YS, Fernstrom A, Barbour S, Sperati J, Villanueva AR, Wu MJ, Wang D, Borodovsky A, Badri P, Yureneva E, Bhan I, Cattran DC. FR-OR67 Exploratory Results from the Phase 2 Study of Cemdisiran in Patients with IgA Nephropathy. *ASN Kidney Week 2022* 2022.

104. Lafayette RA, Rovin BH, Reich HN*, et al.* Safety, Tolerability and Efficacy of Narsoplimab, a Novel MASP-2 Inhibitor for the Treatment of IgA Nephropathy. *Kidney Int Rep* 2020; **5:** 2032-2041.

105. Barratt J RB, Zhang H, Kashihara N, Maes B, Rizk D, Trimarchi H, Sprangers B, Meier M, Kollins D, Wang W, Magirr A, Perkovic V. POS-546 Efficacy and Safety of Iptacopan in IgA Nephropathy: Results of a Randomized Double-Blind Placebo-Controlled Phase 2 Study at 6 Months. *Kidney International Reports* 2022; **7**.
